# Management of Lipedema with Ketogenic Diet: 22-Month Follow-Up

**DOI:** 10.3390/life11121402

**Published:** 2021-12-15

**Authors:** Roberto Cannataro, Sandro Michelini, Lorenzo Ricolfi, Maria Cristina Caroleo, Luca Gallelli, Giovambattista De Sarro, Alberto Onorato, Erika Cione

**Affiliations:** 1 Galascreen Laboratories, University of Calabria, 87036 Rende, CS, Italy; rcannataro@nutrics.it (R.C.); mariacristinacaroleo@virgilio.it (M.C.C.); 2 Department of Pharmacy, Health and Nutritional Sciences, University of Calabria, 87036 Rende, CS, Italy; 3 San Giuseppe Hospital Marino, 00047 Rome, Italy; sandro.michelini@aslroma6.it; 4 Villa Hesperia, 18012 Bordighera, IM, Italy; lorenzoricolfi@hotmail.it; 5 Clinical Pharmacology and Pharmacovigilance Operative Unit, Department of Health Science, University of Magna Graecia, Mater Domini Hospital Catanzaro, 88100 Catanzaro, CZ, Italy; gallelli@unicz.it (L.G.); desarro@unicz.it (G.D.S.); 6 Linfamed, 33100 Udine, UD, Italy; a.onorato@linfamed.it

**Keywords:** lipedema, inflammation, ketogenic diet, pain management, low carb

## Abstract

Lipedema is a pathology of adipose tissue, still of unclear etiology and challenging to diagnose. For these reasons, a therapeutic approach is also complex and sometimes controversial. The inflammation state present in lipedema can be limited by controlling the glycemic peaks. Specifically, the ketogenic diet (KD) seems to have the right conditions to be effective. Herein, we reported a subject diagnosed with lipedema who, with only KD nutritional intervention, achieved a significant weight loss (−41 Kg), with a net decrease in body circumferences, and also reporting an improvement in pain, and therefore in the overall quality of life. She refused other types of intervention and kept KD for two years. This case could represent the first step to organize a KD nutritional protocol specifically applied to lipedema.

## 1. Introduction

Lipedema is a chronic pathology of the subcutaneous adipose tissue that was defined for the first time by Allen and Hines in 1940 [1]. The diagnosis is not yet well standardized, nor is the etiopathogenesis. Consequently, the treatment of this condition also is not unanimous. In any case, it should be examined from a holistic point of view, together with the treatments typically used, which include: (i) surgery; (ii) compression garments; and (iii) physiotherapy. Today, special nutrition is not considered a treatment of lipedema. However, it should have an important place, because it could represent a way, in the long term, to manage the inflammation—mostly subclinical—that is almost always present [2] in this condition, which almost exclusively affects the female population, and so it is considerate gender-related [3,4]. There is undoubtedly a genetic component, as already hypothesized by Herbst et al. [5], and partially confirmed by the study of Polacci et al. [6], which highlighted the mutation of the gene AKR1C1. Generally, lipedema begins following significant hormonal changes, which is why, in addition to the genetic predisposition mentioned above, the pathophysiological process based on hormonal alterations is mainly associated with estrogen receptors (ERs) in the adipose tissue, which are abnormally expressed. ERs exist as alpha and beta (ER-α and ER-β) [7,8]. It has been hypothesized that in lipedema, there is an unbalance between ERs (ER-α is downregulated and ER-β is upregulated) in the areas of the adipose tissue affected. At the same time, microangiopathy resulted in a minimal, but constant, tissue hypoxia, with an increase in capillary permeability, responsible for transient oedema aggravated by prolonged orthostasis, and a marked tendency to form hematomas and petechiae, all associated with dysregulation of the veno-arteriolar reflex [8,9]. The typical pain of lipedema could derive from the repercussions of tissue hypoxia associated with a locoregional alteration of the sensory nerve fibers or tissue compression of these fibers, with consequent inflammatory state of the affected part [9,10]. The skin appears fresh, pale blue, soft, and treatable; Stemmer’s sign is negative [7,8,9,10]. Several types and stages are used to characterize lipedema [7,8], and in this enigmatic disease of the peripheral adipose tissue [10,11], patients display several comorbidities, including migraine [12]. The common thread of nonsurgical management of lipedema should be to limit possible inflammatory states. In this view, anecdotally, the dietetic approach applied to lipedema is the rare adipose disorders (RAD) diet, which includes, among others, the exclusion of red meat, milk derivatives and sweeteners, but in the current state of things, there is no reason to exclude these or other foods. It should be emphasized, instead, that if intolerances occur (for example, nonceliac gluten sensitivity, lactose, or nickel, to name the most frequent), it is necessary to strongly limit these categories of foods, in order to not accentuate the inflammatory state already present.

The only work published at the moment concerns a nutritional approach inspired by the Mediterranean diet (rich in fruit and vegetables), and features a lower carbohydrate intake than usual of around 40% of the total caloric intake [13]; this approach proved to be partially effective, most likely thanks to the glycemic control (low carbohydrates and good fiber ingestion) and the simultaneous intake of vitamins and polyphenols, which are typical of the Mediterranean diet, with a proven epigenetic effect [14].

The ketogenic diet (KD) has an interesting rational application, both for the regulation of blood sugar and for an anti-inflammatory effect, but it lacks a study on a large sample. It should be pointed out that nutritional programs for lipedema, even if overweight or obesity is present, must not have the sole objective of weight loss, as programs of this type often result in failure both in the management of lipedema and weight loss, also creating frustration in affected subjects.

In this latter view, our group recently demonstrated that KD had positive effects on neuroinflammation and antioxidant status in obese subjects [14]. Herein, a collection of 22 months of follow-ups of a subject with lipedema who kept a KD is reported.

The aim was to confirm our hypothesis on the effectiveness and safeness of the KD in this pathophysiological condition, even when kept for long time [15,16]. The KD is a nutritional program that provides for a minimal daily intake of carbohydrates (<30 g or <10% of the total calories), and has been used for almost 100 years to manage drug-resistant forms of childhood epilepsy [17]. To date, it is proposed in support of cancer therapy by exploiting the Warburg effect [18,19], in the management of migraines [20,21], in polycystic ovary syndrome [22], and obviously for weight loss, in particular in obese subjects [23,24]. It is now well established that if operated under specialist supervision, it is safe, even if kept in the long term [24]. Lastly, studies on KD highlighted an epigenetic action on DNA [25,26,27] and miRNAs (powerful regulators of protein synthesis) [27,28]. The main features of the KD applied for lipedema could be the anti-inflammatory and the regulatory actions in the management of free radicals [26,27,28,29,30].

## 2. Case Presentation

### 2.1. Data Collection and Questionnaires

The patient was a 32-year-old woman diagnosed with lipedema type IV and V, stage II–III; she complained of widespread pain, particularly in the lower limbs; heaviness; and difficulty in making various movements. She refused any type of treatment if it was not nutritional. An assessment of pain and quality of life was carried out through the Western Ontario and McMaster Universities Arthritis Index (WOMAC) [31], Sleep Quality Scale (SQS) [32], RAND-36 [33], and VAS [34] questionnaires.

### 2.2. Bioimpedance Analysis

Bioimpedance analysis (BIA) [35,36] was performed with a bioimpedance analyzer (BIA 101 Anniversary, Akern, Florence, Italy) using a phase-sensitive device with an alternating current at a frequency of 50 kHz. The accuracy of the BIA instrument was validated before each test session, following the manufacturer’s instructions. Measurements were made on a medical bed isolated from electrical conductors. The subjects were in the supine position with legs (45° compared to the median line of the body) and arms (30° from the trunk) abducted. After cleansing the skin with alcohol, two electrodes were placed on the right hand and two on the right foot. Resistance (R) and reactance (Xc) parameters were divided by standing body height in meters. Phase angle (PhA)was calculated as the arctangent of Xc/R × 180°/π. Body fat (Bf) was calculated with Bodygram software.

### 2.3. Nutritional Plan

We choose to operate a caloric deficit of 200–250 Kcal compared to the 14-day food diary reported, as we did in a previous work [37] to better design the nutritional program; the carbohydrate intake was set at no more than 25 g per day. The ratio between proteins and fats was between 1:1 and 1:2; the choice of having a considerable protein intake was to preserve the muscle mass as much as possible, which was already in suboptimal conditions, probably also due to lipedema. No kind of food was excluded; therefore, milk and milk derivatives, red meat, and even gluten were included (the latter in any case in small quantities, as it is related to carbohydrates). We immediately inserted nutritional supplements: omega3 fish oil (3 g per day of product, about 1.8 g of DHA + EPA), vitamin C (1 g per day divided into 2 doses), and vitamin D (2000 iu per day); all supplements were from 4+ Nutrition, Padua—Italy. Table 1 shows an example of a daily meal. The program provided on average an intake of 1300 Kcal, divided into 30% from proteins, 66% from fats, and 4% from carbohydrates. The recommended sources of protein were mainly meats (veal, pork, chicken, turkey) and fish (of any type with even more fat, such as salmon, anchovies, or mackerel) or seafood, eggs and dairy products. Vegetables, preferring those in season and limited to those with a non-negligible carbohydrate content, such as eggplant, peppers and tomatoes, were included. Obviously, we completely prohibited tubers, squash, legumes, and any type of cereal or pseudocereal (unless they fell within the small amount of carbohydrates allowed; for example, 30 g of rye bread in the morning). The sources of fat were mainly extra virgin olive oil (butter or mayonnaise was granted as a substitute, but these choices were unwelcome, therefore little used) and nuts or seeds. The patient had continuous support via text messaging via mobile phone, and we saw her every month in our laboratory.

The patient also was given indications and suggestions on how to cook food, also with few limitations in this case; for example, even fried food was allowed in moderation. After the sixth month, we included a fortnightly free meal, which included a quantity of carbohydrates ranging from 60 to 120 g; then after the twelfth month, the free meal was offered every week, and this did not affect the progress of the program itself. We must say that during this long period, there were some moments in which the program was not followed in an optimal way; for example, during the Christmas period, we could not monitor the changes except to report a flare-up of the pain reported that then subsided as soon as the program was reinstated.

### 2.4. Ketosis Assessment

We checked ketosis status every week (self-administered) via Bayer Keto-diastix (Bayer—Germany) and monthly via a Wellion Galileo Glu/Ket blood glucose meter (Wellion—Austria). The latter showed a value higher than 0.8 mmol/L in every test.

### 2.5. Side and Unwanted Effects of the KD

We checked the following side effects during the nutritional plan of the KD: frequent urination, fatigue, hunger, confusion, anxiety and/or irritability, and sweating. The unwanted effects included: constipation, headache, difficulty in focusing, and muscle soreness.

## 3. Results

**Figure 1:**
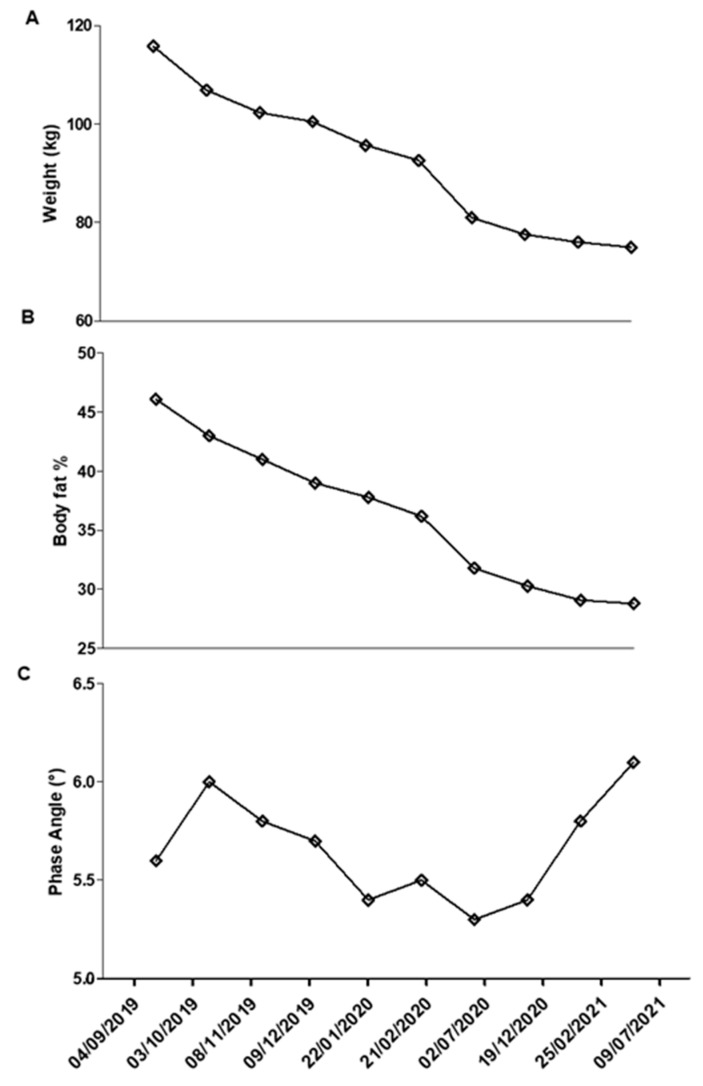
(**A**) Weight; (**B**) body fat percentage; and (**C**) phase angle recorded during 18 months.

**Figure 2:**
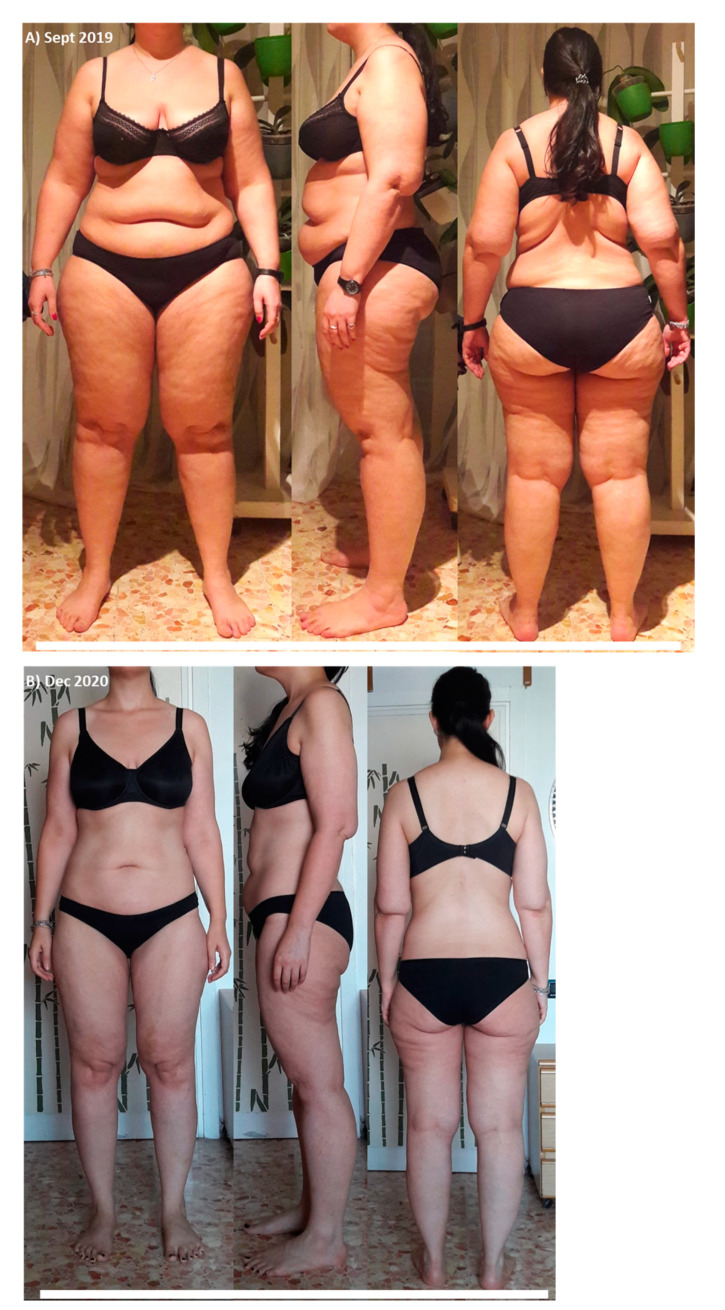
(**A**) Before starting ketogenic diet in September 2019; (**B**) after 14 months of ketogenic diet.

The results in ponderal terms were striking: the subject lost 41 kg (Figure 1A), with a change of about 20 Bf%, as shown in Figure 1B, and maintained a healthy condition, as evidenced by the phase angle (Figure 1C). Any side effect was recorded, and only a difficulty in focusing at day 3 that returned to normal at day 5. Visible changes are apparent between Figure 2A,B; it can be seen that in addition to important weight loss, some other points typically affected by lipedema, including arms, thighs, and abdomen, were improved.

The patient, at the beginning and every seven months afterward, underwent biochemical blood tests to monitor the following parameters: glycaemia, insulin, glycated hemoglobin (HbA1C), vitamin D, hemoglobin (Hb), iron, calcium, potassium, aspartate aminotransferase (AST), alanine aminotransferase (ALT), gamma-glutamyl transferase (GGT), creatinine, c-reactive protein, and ureic acid. Blood tests were performed at an accredited clinical biochemistry laboratory while the patient was in a fasted state. Vitamin D increased and insulin decreased, as shown in Table 2. Even HOMA-IR was greatly affected.

However, despite being a very valid result, this was considered secondary to the impact on lipedema, as can be from the body measurements shown in Table 3. Body circumferences showed a decrease in all districts: 37.5 cm less on the hips, and 23.9 cm less on the waist; however, it should be emphasized that there were also significant decreases in body areas typically affected by lipedema, such as arms (−10.5 cm left, −11.5 cm right), forearms (−6.5 cm both), knees (−8.5 cm both), calves (−9 cm left, −8.5 cm right), and ankles (−2.5 cm left, −3 cm right).

**Figure 3:**
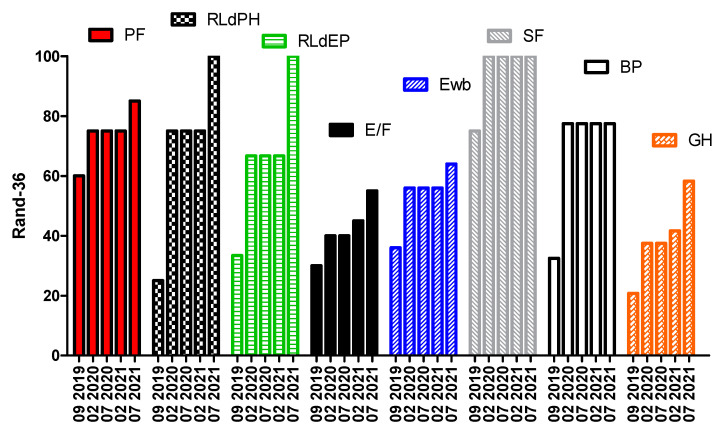
RAND-36 questionnaire results. PF: Physical Functioning; RLdPH: Role Limitations due to Physical Health; RLdEP: Role Limitations due to Emotional Problems; E/F: Energy/Fatigue; Ewb: Emotional well-being; SF: Social Functioning; BP: Body Pain; GH: General Health. Taken together, these represented a great improvement in the overall quality of life.

In the four questionnaires (RAND-36, WOMAC, SQS, and VAS) administered during the period, a clear improvement was shown in the quality of life, even in daily actions; in the quality of sleep; and also in the perception of the pathology as a more manageable, albeit limiting, condition, as shown for RAND-36 in Figure 3.

The WOMAC score had a decrease of 53%, and SQS 48%. We could not make a direct measurement of pain, but from the VAS scale, it was evident that the overall improvement was 67%, up to a condition of normality (Table 4).

## 4. Discussion

The first part of the program lasted more than one year, from September 2019 to July of 2021. The patient did not report any particular side effects, except in the very first days of adaptation to ketosis, and therefore good intestinal regularity, better mental freshness, and the possibility of performing normal activity. She sometimes experienced halitosis, but also tranquility; she was in contact with other patients suffering from lipedema and on a ketogenic diet, in particular thanks to the support of the Italian Association of Lipedema (LIO). The most significant result, in our opinion, was the strong compliance, which allowed the subject to continue the program even during the first, unexpected lockdown due to the COVID-19 pandemic. This also was thanks to a variety in food choices, as no food category was excluded, confirming that there was no food that directly influenced lipedema unless specific intolerances or allergies were manifested. The excellent results highlighted by the questionnaires could be confirmation of the anti-inflammatory effect of the ketogenic diet. In the first period, the patient practiced physical activity independently; the program included 90–120 minutes of brisk walking per week and 1–2 free bodyweight sessions of around 30 minutes at low intensity. Due to work commitments, the subject was not always able to complete the entire program; however, she did not manifest decreases or difficulties due to calorie restriction or diet in general. The program was completely interrupted during the lockdown period (in fact, a worsening of the phase angle can be seen), then resumed in particular in the last period; this underlines how organized physical activity was desirable to promote the maintenance, if not the improvement, of the muscular condition (already limited by the lipedema itself). It should be emphasized that in order to better manage the condition of lipedema, this point should also be considered under the supervision of an expert in motor activity.

The biochemical–clinical parameters showed that renal and hepatic function were not negatively affected; on the other hand, basal insulin decreased significantly, moderating the insulin resistance present in the initial state evidenced by the HOMA-IR index (unfortunately, it was not possible to administer a glucose/insulin tolerance test). CRP was decreased, even if not elevated from the beginning, which could be a sign of less inflammation (a future work objective will be to monitor cytokines and other inflammatory markers such as miRNAs).

As already pointed out above, the scientific literature on the lipedema–nutrition link is scarce; we would like to see this case report as a starting point to organize trials, certainly with a high number of subjects, and perhaps comparing different nutritional schemes; for example, in an approach that limits glycemic peaks, but that does not lead to ketosis. It would be very interesting to evaluate inflammatory cytokines and/or CRP; test the pain directly and not only through questionnaires; test the miRNAs, which are modulated by diet, in particular the ketogenic diet [25], but which could be characteristic of lipedema [37], in order to evaluate its evolution, or even create a diagnostic tool.

We think that the ketogenic diet could be a very useful tool to consider in the management of lipedema; certainly this was a particularly favorable case, as in other situations, there are different aspects, but also less compliance (this aspect could be moderated by alternating periods of a ketogenic diet with periods of a low-carb diet; i.e., with an intake of carbohydrates around 100–150 g per day—we in fact are working on a program of this type on a large scale). The cost of the program, as pointed out by Kabisch et al. [38], was not a secondary factor, even in the specific case; perhaps also thanks to the good results, it was not a problem.

Obviously, we do not want to propose the ketogenic diet as the only possible option both from a nutritional point of view and in general for the treatment of lipedema. It must be considered as a set of interventions to be effective and long-lasting as recently highlighted [39], not only thanks to the expected glycemic regulation, but also thanks to the anti-inflammatory action [25,39].

## Figures and Tables

**Table 1:** Example of the KD nutritional plan.

Nutritional Plan	
Breakfast	30 g of rye bread
	40 g of ham
	20 g of spreadable cheese
	1 espresso coffee without sugar or other sweetener
Snacks (during the day)	50 g of parmesan cheese
	20 g of nuts (walnuts, hazelnuts, cashews, almond)
	40 g of chicken sliced breast
	Vegetables at will (from a list that considered a limited intake of carbohydrates)
Lunch	200 g of salmon
	A large bowl of mixed salad
	2 tablespoons of extra virgin of olive oil
	Vinegar and spices at will
Dinner	1 whole egg and 100 mL of pasteurized egg white
	A large bowl of grilled vegetable (zucchini, eggplant)
	2 tablespoons of extra virgin of olive oil
	Vinegar and spices at will
Drink at least 2 L of water, with carbonated sweetened beverages permitted	

**Table 2:** Biochemical parameters monitored during the program.

Biochemical Parameters	Baseline	7th Month	14th Month	21st Month	Normal Range
Glycemia mg/dL	99	95	92	90	70–100
**Insulin mIU/L**	**29.3**	**14**	**10**	**11**	**4–25**
**HOMA-IR**	**7.16**	**3.28**	**2.27**	**2.44**	**<2.60**
HbA1C mmol/mol	32	26	25	26	20–38
**Vitamin D ng/mL**	**15**	**30**	**32**	**32**	**30–50**
Hb g/dL	13.9	13.2	13.7	13.5	12.0–15.5
Serum iron µg/dL	102	89	98	95	60–170
Serum calcium mmol/L	2.4	2.2	2.29	2.3	2.2–2.7
Serum potassium mmol/L	3.74	3.8	4.39	4.01	3.6–5.2
AST U/L	21	19	19	9	8–33
ALT U/L	21	16	13	12	4–36
GGT U/L	16	12	13	12	0–30
Creatinine mg/dL	0.93	0.87	0.85	0.72	0.6–1.2
CRP mg/dL	0.6	0.2	0.1	0.1	0.3–1.0
Uric acid mg/dL	2.5	3.5	3	3	2.7–7.3

**Table 3:** Body measurements: report of the body circumferences found during the program.

Circumference	09 2019	10 2019	11 2019	12 2019	02 2020	04 2020	06 2020	08 2020	10 2020	12 2020	D%
Arm sx	43	42	40.5	39.5	39	38.5	36	36	35	32.5	−24.42
Arm dx	44	42	40.5	40	40	38	37	36.5	35	32.5	−26.14
Forearm sx	32	31	30	29	28	28	27	27	27	25.5	−20.31
Forearm dx	32	30	30	29	29	29	26	27	27	25.5	−20.31
Waist	109	99.5	96.5	96	96	94	90	88.5	86	83	−23.85
Hip	134	123.5	116	105.5	103.5	102	104	102	101	96.5	−27.98
Coulotte	133	132	127.5	124	120	118.5	115.5	115	111	107	−19.55
Thigh sx	74	71.5	69.5	66	62	59.5	56.5	56.5	56	54	−27.03
Thigh dx	74	71.5	69.5	67	64.5	61	58	57.5	56.5	54	−27.03
Knee sx	47.5	47	46	44.5	43	41	40	42	42	39	−17.9
Knee dx	48	47	46.5	44.5	44	42	42	42	42	39.5	−17.71
Calf sx	50.5	48	47	46.5	46	45.5	44	44	43	41.5	−17.82
Calf dx	50	48.5	48	46	46	44.5	44	44	43	41.5	−17
Ankle sx	26	25	25	24,5	24	24	23.5	24	23.5	23.5	−9.62
Ankle dx	26.5	25	24.5	24.5	24	24	23.5	24	23.5	23.5	−11.32

**Table 4:** Other questionnaire results.

Time	WOMAC	SQS	VAS
09 2019	45	37	9.2
02 2020	38	26	7.1
07 2020	37	24	6.7
02 2021	35	22	3.5
07 2021	21	19	3
D%	−53.33	−48.65	−67.39
